# Calcium and Its Compounds

**Published:** 1861-12

**Authors:** 


### DENTAL REGISTER OF THE WEST.

VOL. XV.] DECEMBER, 1861. [No. 12.

### Original Essays and Communications.

### CALCIUM AND ITS COMPOUNDS.

CALCIUM, Ca=20.—From force of habit we are apt to regard the metals as derived solely from the inorganic kingdom of nature. The one now under consideration, however, like several others, is found in both divisions of the organic. This metal, indeed, performs no inconsiderable part in the great play of life; and, when this is borne in mind, an intimate acquaintance with it and its compounds can not be otherwise than interesting.

Though so abundant and so extensively diffused, it is not found uncombined. Its strong affinity for the highly electro-negative elements, such as oxygen and fluorine, and the difficulty with which it is separated from them, explains the fact of its recent discovery. Its existence was unknown till the early part of the present century.

Calcium is a yellowish white metal, having the color and luster of gold heavily alloyed with silver. Its luster is soon tarnished by the oxygen of the atmosphere, especially in damp air, when its yellowish tinge is more perceptible. It is ductile and malleable, and about as hard as silver. It burns with a bright flash, when heated on platinum; and, when heated, burns brilliantly in oxygen, chlorine, iodine, sulphur, etc. It decomposes water rapidly, with the evolution of hydrogen and the formation of hydrate of lime. Calcium is usually obtained by decomposing its chloride by galvanic action. Its specific gravity is about 1.58; and it melts only at a high temperature. On account of its strong affinities, the metal is of no use in the arts; and as there are other and more convenient deoxydizers, it is but little used in the laboratory.

LIME, $CaO=28$.—Calcium forms two compounds with oxygen, the protoxyd and the binoxyd. The former is called lime, and is extensively used both in the laboratory and the arts. It is commonly obtained by heating the carbonate of lime to redness in lime-kilns. For use in the laboratory it should be prepared from calcareous spar, or fine marble, by heating in an open crucible. When thus prepared it consists of porous masses of a white color, sufficiently cohesive to bear transportation, and two or three times as heavy as water. Lime is infusible in the furnace, and but partially fusible in the oxyhydrogen flame. It is a strong basic oxyd, and turns reddened litmus blue. It has a strong affinity for water, and is used to separate this liquid fiom alcohol and kindred substances.

HYDRATE OF LIME, $CaO, HO=37$.—When water is thrown upon massive lime, as prepared above, it is first imbibed, as it would be by other porous bodies, but soon unites chemically with it, forming a new compound. The lime falls to powder, greatly increased in bulk, and is then said to be slaked. And it is worthy of notice that though twenty-eight parts of lime have united with nine of water, there is no moisture present. By combination, the water has become a part of a dry solid. And, in accordance with a universal law, that when a liquid is condensed into a solid, latent heat is liberated, and an increase of temperature is the result, by the slaking of lime, sufficient heat is evolved to char wood, and even to produce actual ignition. By the slaking of one equivalent, or twenty eight parts of lime, as much heat is liberated as would raise the temperature of one part of water 1212°.

Hydrate of lime is slightly soluble in water, and is one of the few substances more soluble in cold than in hot water. One grain of the hydrate is held in solution by 778 grains of water at 60°, while it requires 1270 grains of boiling water to accomplish the same result. By slowly evaporating the solution in vacuo, small transparent hexahedral crystals of the hydrate may be obtained.

Limewater is readily prepared by adding an excess of the hydrate to distilled, or clean rain water, and repeatedly agitating the mixture for a few days. When left to settle, the excess falls to the bottom, and the clear solution may be carefully decanted, or drawn off with a syphon.

Lime-water absorbs carbonic acid from the atmosphere, and becomes covered with a thin pellicle of carbonate of lime. A portion of this carbonate also falls to the bottom of the vessel, in the form of powdered chalk When hydrate of lime is for a long time exposed to the atmosphere, a half an equivalent of carbonic acid is absorbed by it, and a definite compound of the carbonate and hydrate is formed, as represented by the formula, $CaO, CO_2 + CaO, HO$.

Lime-water is often a valuable remedy for an acid state of the secretions. Often when the local use of alkaline carbonates fails to arrest an acid state of the secretions of the mouth, the internal use of lime-water, for a few days or weeks, acts like a charm. We usually prescribe it in teaspoonful doses three times a day, after eating, though, of course, the dose must be varied to suit the age of the patient and the peculiarities of the case. And, as most dentists are not physicians, it will be advisable to act through, or obtain the consent of the family physician. The taste of lime-water is too harsh and acrid to admit of its use as a local remedy, in acidity of the mouth; but this is not to be regretted while the carbonate of lime—prepared chalk—accomplishes all that it could.

Carbonate of Lime, $CaO, CO_2 = 50$.—This salt, sometimes pure, but often commingled with other substances, is found abundantly in nature. It exists in the forms of calcareous spar, marble, chalk, limestone, etc. As thus found, the salt is anhydrous; but a hydrated carbonate of lime may be obtained, by slightly heating together 1 part of hydrate of lime, 6 parts of water, and 3 of sugar, filtering the solution, and exposing it, for a week or two, to the atmosphere, in a shallow vessel. The carbonic acid is obtained from the atmosphere; and the hydrated salt crystallizes with five equivalents of water. The crystals are acute rhombohedrons.

Carbonate of lime is nearly insoluble in pure water, but dissolves, to a considerable extent, in water charged with carbonic acid. On this account it is generally present in the water of wells and springs, and is precipitated from it by boiling, which expels the excess of carbonic acid. The same result takes place when the water is evaporated at low temperatures, or even when it is exposed to the atmosphere. For example, when the water flows over wood, or other destructible substances, the forms of these substances are preserved in calcareous deposits of carbonate of lime. When a current of carbonic acid is forced through lime-water, the lime is precipitated in the form of powdered carbonate; and if the supply of the acid is kept up, most of the powder will be redissolved.

On account of the elasticity of carbonic acid, the carbonate of lime (as well as other carbonates) is easily decomposed by the application of heat, or a less volatile acid. In the ordinary preparation of lime, the heat simply drives off an equivalent of carbonic acid. If, however, the carbcnate be hermetically sealed in an iron tube, and heated, it may be fused without undergoing decomposition. If the tube is cooled slowly, the salt crystallizes, and resembles marble.

Carbonate of lime performs important offices in the animal economy. It is an essential constituent of the bony tissues, and the principal ingredient of the shells of mollusks. As a constituent of the teeth, it is interesting to the dentist. All acids capable of acting on the calcareous portion of the tooth, decompose the carbonate, liberating the carbonic acid. In other words, the action of an acid on this salt in the tooth, differs, in no respect, from its action on the same salt in any other situation. Hence, with hydrochloric acid, chloride of calcium is formed, with sulphuric, sulphate of lime, and so on, through the entire catalogue of acids capable of corroding the teeth.

It would be interesting to inquire how this salt is introduced into the system. True, most men and other animals drink water strongly impregnated with it; but it is quite probable that much of it is formed from other salts of lime, introduced with vegetable food. It is the opinion of most physiological chemists that free carbonic acid may be found in all the animal fluids; and, if this be true, they would have no difficulty in holding the carbonate in solution, ready for assimilation.

SULPHATE OF LIME, $CaO, SO = 68$.—When sulphuric acid is added to any soluble salt of lime, this salt is precipitated, as a bulky white powder. It is also found in nature, in two conditions. When composed as indicated in the above formula, it is called *anhydrite;* but it is found more abundantly in combination with water, as represented in the formula, $CaO, SO + 2\ HO$, when it is called *gypsum*, or plaster of Paris. Gypsum, or the hydrated sulphate, is more important and interesting to the dentist than the anhydrous.

Sulphate of lime is but slightly soluble in water, nearly five hundred grains of the liquid being required to dissolve a single grain of the salt. It fuses at a strong red or white heat, without decomposition; but when thus heated in contact with charcoal, its oxygen is all expelled, and the protosulphide of calcium, $CaS$, is formed, which is a whitish salt, nearly insoluble in water.

The native gypsum is broken into small fragments, and heated in ovens or kilns, to a temperature of 240° to 260°, and afterward reduced to powder. It is then the "plaster" of our laboratories. When heated to 300°, or with some specimens, even to 270°, it parts with all of its water, and fails to recrystallize when mixed with water, and, therefore, becomes useless in the laboratory.

Much of the gypsum found in nature is impure. That found in the vicinity of Paris, according to Regnault, contains,

| | |
|---|---|
| Sulphate of lime | 70.39 |
| Water | 18.77 |
| Carbonate of lime | 7.63 |
| Clay | 3.21 |
| | 100.00 |

Other foreign substances are found in some specimens, which may account, to some extent, for the imperfect recrystallization of some varieties of plaster. The gypsum must have the proper composition, and must be properly calcined, in order to obtain a perfect plaster. When thus prepared, if the plaster is mixed with water, to the consistence of a thin paste, a chemical union takes place between the water and the sulphate, by which the paste is converted into a solid mass of gypsum. This crystallization is commonly called *setting.* In setting, plaster expands slightly, and is thus forced into all the inequalities of an ordinary mould. The degree of expansion differs with different varieties. The experiments of Prof. Buckingham, as reported in the Dental *Cosmos*, are the most satisfactory of any we are aware of, with reference to this expansion, and we refer the curious to them. It is satisfactory to know that it is of no practical importance in the dental laboratory.

When plaster sets, the combining water is condensed from a liquid to a solid; and, of course, latent heat is liberated. The consequent elevation of temperature is quite perceptible, but is less than when hydrate of lime is formed.

Pure crystallized gypsum is sometimes called *selenite;* and a white compact variety of it, used in statuary ornaments, is called *alabaster.*

The various uses of plaster, and the modes of using it in the dental laboratory, belong to the mechanic, rather than to the chemist. Fortunately there is no lack in this direction, and we, accordingly, refer the reader to resources already familiar to him.

PHOSPHATE OF LIME, $3\ CaO, PO_5 = 156$.—Phosphoric acid and lime unite in several proportions; but it is not intended to notice specially, in this connection, any but the salt indicated by the above formula, which is a sub-phosphate, and is commonly called, for sake of definiteness, *bone phosphate.*

Bone phosphate may be formed artificially, by adding a solution of chloride of calcium to a solution of the rhombic phosphate of soda; but as it exists abundantly, ready formed in bones, it is usually obtained from this source. When bones are fully calcined in open vessels, the organic matter is all driven off, and a white ash remains. Bone ashes are usually composed of four-fifths of sub-phosphate, and one-fifth of carbonate of lime. For separating the two salts, the process of the Dublin College is as convenient as any other. The bone ash is reduced to powder, and dissolved in highly diluted hydrochloric acid. When the acid is saturated, the solution is filtered, and ammonia is added as long as a precipitate is thrown down. Afterward, wash and dry the precipitate. The carbonate of lime contained in the ash is decomposed by the action of the hydrochloric acid, as indicated by the following formula,—$CaO, CO + HCl = CaCl + HO + CO_2$.

The carbonic acid, being liberated, escapes as a gas, and the chloride of calcium, being highly soluble, is easily removed by washing. The precipitate is the subphosphate under consideration.

This salt is a white, tasteless, odorless, gritty powder. It is insoluble in water, but dissolves in hydrochloric, nitric, acetic, and lactic acids, without decomposition, and may be precipitated from its solution in any of these acids, by the alkalies or their carbonates. At a very high heat it fuses without decomposing.

As this salt is the principal earthy ingredient of the teeth, an accurate knowledge of its properties and affinities is of vast importance to the dental surgeon. And it is worthy of notice by all that, in the formation of the teeth, and osseous tissues in general, the great Creator selects, as the principal solid constituent, a salt so permanent in its composition—a salt that can sustain white heat, or solution in the most active acids, without change.

In the study of the chemistry of caries, this salt and the carbonate are the only solid ingredients of the tooth necessary to be taken into consideration; for the others are present in such small quantities that they produce no practical or observable results. And it is time that all notions about other acids displacing the phosphoric, and taking the lime, were abandoned; for no acid directly concerned in the production of caries takes the lime from the phosphoric, unless it be sulphuric acid; and it can do so only in favorable circumstances. But they do take the lime, and the phosphoric acid along with it. The direct action of an acid in producing caries, as far as the earthy constituents of the tooth are concerned, is exactly the same as its action on bone ash. It is true the pulverized condition, or porosity of the ash favors the action of the acid, by promoting contact; but if an acid acts at all on the solid constituents of the tooth, it acts precisely as it does on the same substances out of the mouth. Let it be borne in mind, then, that hydrochloric, nitric, lactic and acetic acids, all dissolve the phosphate of lime, that is, they *combine* with it, forming soluble compounds, while the same acids *decompose* its accompanying carbonate. The action of these acids should be understood and remembered, for the teeth are oftener destroyed by them than by any others.

Writers on Therapeutics sometimes express a want of confidence in the efficacy of this salt, as a remedy for a lack of it in the system, on account of its insolubility, apparently forgetting its high degree of solubility in hydrochloric acid, which is present in the gastric juice.

That the phosphate of lime performs important offices in the animal economy, may be inferred from its extensive diffusion through the system. According to Lehmann, "there is no animal tissue in whose ash, or incineration, we do not find phosphate of lime." And the importance of furnishing an abundant supply of it in certain conditions of the system, either in the food, or otherwise, is worthy of the most serious attention; but to pursue this vein would lead beyond the range of the present paper. Lehmann, vol. 1, p. 374, says: "We need hardly remark that rachitis frequently, if not always, occurs simultaneously with the period of dentition, that the consumption of phosphate of lime during pregnancy is often so great that scarcely any traces of it can be found in the urine, and that, during this period of woman's life, fractures unite with extreme difficulty, and sometimes do not unite at all."

The proper consideration of this salt, in all its relations to dental surgery, would require a separate paper.

Hypochlorite of Lime, CaO, ClO=71.—This salt is extensively used in bleaching. When milk of lime, in excess, is added to a solution of hypochlorous acid, it is obtained pure. The commercial chloride of lime, often called *bleaching powder*, is a mixture of hydrate of lime, chloride of calcium, and hypochlorite of lime. It is usually prepared by passing chlorine slowly over hydrate of lime. The reaction which takes place may be represented as follows:

$$2\ CaO+2Cl=CaO,\ ClO+CaCl.$$

An excess of lime must always be present, for if the supply of chlorine is continued after the lime is all changed into chloride of calcium and hypochlorite of lime, the hypochlorite is decomposed, with the formation of chloride of calcium and a chlorate of lime, thus:

$$3\ (CaO,\ ClO)=CaO,\ ClO_5+2\ CaCl.$$

The hypochlorite of lime and the chloride of calcium, being soluble, can be readily separated from the hydrate of lime by washing. According to Graham, ten parts of water take up the bleaching combination of one part of the commercial chloride; but, of course, the proportions must vary, for some specimens contain a much greater excess of the hydrate of lime than others. The solution thus obtained has the odor of hypochlorous acid, an astringent taste, and an alkaline reaction.

It decomposes most organic matters, containing hydrogen, and, therefore, destroys most coloring matters.

Hypochlorite of lime is not a permanent compound. Most of the acids decompose it, taking the lime. The carbonic acid of the atmosphere acts in this way; and the consequence is, that hypochlorous acid is constantly liberated. Even the acid is not permanent, but is slowly decomposed, at ordinary temperatures, with the evolution of chlorine; and this decomposition is greatly facilitated by exposure to light.

In bleaching with this salt, a number of reactions occur. The lime may be regarded merely as the pilot, or engineer, that conducts the acid to the place where its action is desired. Its direct action in promoting or retarding the bleaching process is of no practical importance. Chlorine has long been recognized as the great bleacher; but disputes have arisen as to how it bleaches. Some maintain that, by its affinity for hydrogen, it decomposes water, and the liberated oxygen, with the advantage of its nascent condition, does the bleaching. Others claim that it takes the hydrogen of the coloring principle, and thus bleaches directly. But there is no occasion for dispute; for both positions are correct. And this may enable us to understand the reactions of bleaching by hypochlorite of lime, or, in other words, by hypochlorous acid.

It has been stated that the hypochlorite is constantly giving off the acid; and, also, that the acid itself is decomposed under all ordinary circumstances. Now, bearing in mind that this acid is composed of one equivalent of oxygen, and one of chlorine, by its decomposition, these active elements are simultaneously liberated, having equally the advantage of the nascent state, and, therefore, far more energetic than if previously free, the oxygen spends its force by taking the hydrogen and carbon from the coloring principle, while the chlorine either takes hydrogen from the coloring matter, or from the water present, in which case another equivalent of nascent oxygen is set to work. From this it will be seen that one equivalent of hypochlorite of lime (containing, of course, one equivalent of hypochlorous acid) has as much bleaching power as two equivalents of free chlorine, or two of nascent oxygen.

With these principles understood, the dentist can readily appreciate the action of the hypochlorite in bleaching teeth. It is pretty generally conceded that the coloring principle here is hematin, the composition of which is, $C_{44}H_{22}N_3O_6Fe$. By a single glance at the formula, it will be seen that the chlorine may take its hydrogen, or the oxygen, both its carbon and hydrogen.

The hypochlorite is also valuable as a deodorizer; and it acts, in this direction, by virtue of the same properties and affinities which govern its action as a bleacher.

The remaining salts of lime, such as the nitrate, chlorate, etc., are not possessed of sufficient interest to be noticed here; and as there is no effort at system in this paper, the consideration of them is omitted.

Chloride of Calcium, $CaCl=55$.—By dissolving carbonate or hydrate of lime in hydrochloric acid, chloride of calcium is formed. With the carbonate, the reaction is as follows:

$$CaO, CO_2+HCl=CaCl+H\ O+CO_2.$$

The carbonic acid escapes as a gas; and the chloride may be crystallized by evaporation. No other compound of chlorine with calcium is known. When a solution of this chloride is strongly concentrated by evaporation, and allowed to cool, large crystals of the hydrated salt are deposited, each equivalent of the salt being united with six of water.

The crystallized salt is very deliquescent; and, when mixed with pounded ice (or snow) by virtue of its great affinity for water, both it and the ice are rapidly liquefied, producing an intense degree of cold, sinking the thermometer, according to some experimenters, as low as 49°.

The crystallized salt is valuable in making frigorific mixtures. It is conveniently prepared by saturating diluted hydrochloric acid with chalk, or marble dust, and evaporating the solution till a drop of it, let fall on a marble a porcelain slab, instantly congeals. The whole mass it then cooled, and the crystals reduced to powder, and put into well stopped bottles.

When heated, the hydrated salt fuses in its water of crystallization ; and, at 400°, parts with four equivalents of its water, and forms a porous mass, which has a great affinity for water, and is extensively used for drying gases, and other kindred offices in the laboratory. It fuses at a red heat, having parted with all its water. The fused chloride has a strong affinity for water ; and a large quantity of latent heat is liberated by its combination with that liquid.

Chloride of calcium is among the most soluble of salts ; and as it is one of the salts formed by the action of hydrochloric acid on tooth-bone, it will be readily understood how it is that acid dissolves out the earthy matter, leaving the organic. It is also soluble in, and forms a definite compound with alcohol.

Fluoride of Calcium, $CaF=39$.—This compound is found in nature, sometimes in well defined crystals, and at other times in solid masses of various, and variegated hues. It is often called *fluor spar*. When reduced to powder and heated, it becomes luminous far below a red heat, evolving a violet, or green light. This fluoride fuses at a red heat, without decomposition, and is acted on but slightly by nitric, or hydrochloric acid, but it is decomposed by sulphuric acid, aided by a gentle heat. Its principal use in the laboratory is in the preparation of hydrofluoric acid. It is used in the arts in forming vases and ornaments. W.

